# CXCR7 Functions as a Scavenger for CXCL12 and CXCL11

**DOI:** 10.1371/journal.pone.0009175

**Published:** 2010-02-11

**Authors:** Ulrike Naumann, Elisabetta Cameroni, Monika Pruenster, Harsha Mahabaleshwar, Erez Raz, Hans-Günter Zerwes, Antal Rot, Marcus Thelen

**Affiliations:** 1 Institute for Research in Biomedicine, Bellinzona, Switzerland; 2 Walter-Brendel-Center for Experimental Medicine, Ludwig-Maximilians-University, Munich, Germany; 3 Institute of Cell Biology, University of Münster, Münster, Germany; 4 Novartis Institutes for Biomedical Research, Autoimmunity, Transplantation and Inflammation, Basel, Switzerland; 5 Medical Research Council, Center for Immune Regulation, Institute of Biomedical Research, University of Birmingham, Birmingham, United Kingdom; University of Sheffield, United Kingdom

## Abstract

**Background:**

CXCR7 (RDC1), the recently discovered second receptor for CXCL12, is phylogenetically closely related to chemokine receptors, but fails to couple to G-proteins and to induce typical chemokine receptor mediated cellular responses. The function of CXCR7 is controversial. Some studies suggest a signaling activity in mammalian cells and zebrafish embryos, while others indicate a decoy activity in fish. Here we investigated the two propositions in human tissues.

**Methodology/Principal Findings:**

We provide evidence and mechanistic insight that CXCR7 acts as specific scavenger for CXCL12 and CXCL11 mediating effective ligand internalization and targeting of the chemokine cargo for degradation. Consistently, CXCR7 continuously cycles between the plasma membrane and intracellular compartments in the absence and presence of ligand, both in mammalian cells and in zebrafish. In accordance with the proposed activity as a scavenger receptor CXCR7-dependent chemokine degradation does not become saturated with increasing ligand concentrations. Active CXCL12 sequestration by CXCR7 is demonstrated in adult mouse heart valves and human umbilical vein endothelium.

**Conclusions/Significance:**

The finding that CXCR7 specifically scavenges CXCL12 suggests a critical function of the receptor in modulating the activity of the ubiquitously expressed CXCR4 in development and tumor formation. Scavenger activity of CXCR7 might also be important for the fine tuning of the mobility of hematopoietic cells in the bone marrow and lymphoid organs.

## Introduction

The chemokine system is indispensable for leukocyte trafficking. Chemokines attract immune cells via their cognate G-protein coupled receptors (GPCRs) to sites of inflammation and mediate homing to lymphoid organs [1]. Among the various constituents of the chemokine system CXCL12 (SDF-1) and its receptor CXCR4 possess exceptional properties as they control processes outside of the hematopoietic system. Genetic deletion of either molecule leads to a similar phenotype in mouse embryos [2]–[4] that is characterized by defective lymphopoiesis and myelopoiesis, imperfect vasculature, abnormal brain and heart development leading to perinatal death. These findings led to the assumption that CXCR4 and CXCL12 represent a monogamous receptor-chemokine pair.

Recently RDC1 (CXCR7) was described as second receptor for CXCL12 [5]. CXCR7, a heptahelical receptor with strong phylogenetic similarity to GPCRs [6], was shown to bind CXCL12 and CXCL11 with high affinity, but fails to induce typical chemokine responses, such as cell migration and associated intracellular signal transduction [7], [8]. Nonetheless, mice lacking CXCR7 die perinatally due to heart valve malformation, a phenotype that is recapitulated in mice conditionally deficient for CXCR7 in the endothelium [9]. In humans, CXCR7 is expressed in embryonic neuronal and heart tissue, in hematopoietic cells and activated endothelium [7], [8], [10]. Elevated levels of CXCR7 correlate with the aggressiveness of human prostate cancer and promote growth and metastasis of various mouse tumor models [11]–[13]. Some studies suggest active signaling through the receptor [13], [14] or modulation of CXCR4-signaling through heterodimerization with CXCR7 [9], [15]. However, the biochemical mechanisms behind these effects are not well understood.

Additional evidence for a critical role of CXCR7 in embryogenesis arose from investigations on zebrafish development. The receptor is required for efficient migration of different cells such as the posterior lateral line primordium [16], [17], motoneurons in the hindbrain [18] and the primordial germ cells [19]. Interestingly, the zebrafish genome contains two genes coding for CXCR7a and b and also two genes coding for CXCL12a and CXCL12b. However, expression patterns and functional studies suggest that only CXCR7b and CXCL12a are involved in primordial germ cell migration [20], [21]. While Valentin et al. [17] propose active signaling through CXCR7 for the migration of the lateral-line primordium, Boldajipour and colleagues suggested that CXCR7b expressed in somatic cells facilitates the CXCL12/CXCR4-mediated migration of primordial germ cells by controlling the level of the chemokine in the environment, thereby contributing to the formation of a chemotactic gradient [19]. Similarly, in the case of migration of the lateral line primordium, CXCR7b expression at the back of the cell cluster was suggested to be responsible for reduction in the level of CXCL12a at that location and to contribute to the generation of a chemotactic gradient despite the uniform expression of *cxcl12a* RNA [16].

In the present study we unveil the mechanism of action of CXCR7 in mammalian cells. We observed that CXCR7 cycles between the plasma membrane and endosomal compartments both in the presence and the absence of ligand. In the presence of ligand CXCR7 surface expression is biphasic showing only a moderate reduction of its cell surface levels after prolonged incubation, and is accompanied by marked uptake and degradation of CXCL11 and CXCL12. By contrast, CXCR4 becomes permanently down-regulated following stimulation with CXCL12 and its internalization proceeds with a minimal degradation of the chemokine. Furthermore, we show CXCR7-mediated sequestration of CXCL12 by the endothelium of mouse heart valves and of human umbilical veins. Taken together, our observations support the notion and provide mechanistic insights concerning the role of CXCR7 as a specific scavenger for CXCL12.

## Methods

### Ethics Statement

Human umbilical cords were obtained from the local hospital (Ospedale San Giovanni, Bellinzona) with confirmed verbal consent of the donors. The procedure was approved by the local ethics committee Comitato Etico Cantonale, CH-6501 Bellinzona. Animals were bred and treated in accordance with the Swiss Federal Veterinary Office guidelines. Experiments were approved by the “Dipartimento della Sanitá e della Socialitá”.

### Cell Culture

Madin-Darby canine kidney (MDCK) and HeLa cells were grown in complete Dulbecco's modified Eagle's medium (DMEM) supplemented with 10% fetal bovine serum (FBS), penicillin and streptomycin. Raji B cells were cultured in RPMI 1640 medium supplemented with 10% FBS, GlutaMAX™-I, sodium pyruvate, non-essential amino acids, penicillin and streptomycin (all Invitrogen). Human umbilical vein endothelial cells (HUVECs) prepared and grown as described [22] were kindly provided by B. Wolff and used at passages 3–4.

### Plasmids and Transfections

A CXCR7-DRYLAIV mutant was generated by introducing site-specific point mutations at amino acids Ser(145) and Thr(147) of human CXCR7 by PCR (AccuPrime™ Pfx SuperMix, Invitrogen) with the following primers: 5′–CCGCTACCTCGCCATCGTCTACTTCACC, 5′–GGTGAAGTAGACGATGGCGAGGTAGCGG. The myristoylation/palmitylation motif from the Lck tyrosine kinase was attached to the N-terminus of mCherry to generate a plasma membrane marker [23]. All mutations were confirmed by DNA sequencing. Cells were transfected with pcDNA3 plasmids encoding the human wild type and mutated receptors using Lipofectamine™ 2000 (Invitrogen), followed by G418 (Sigma-Aldrich) selection. Surface receptor expression levels were determined by FACS analysis using saturating amounts of primary antibodies (15 µg/ml anti-CXCR4 (12G5) and anti-CXCR7 (9C4)) and secondary antibodies (10 µg/ml Alexa Fluor 488-F(ab′)_2_ goat anti-mouse IgG).

### Receptor Expression on Cell Surface after CXCL12 Incubation

Daudi B cells were incubated in RPMI culture medium containing 0, 100 or 1000 nM CXCL12, 50 µM cycloheximide, and washed briefly with 1 M NaCl, 50 mM glycine, pH 3. When indicated 2 µM bafiloycin A1 (Sigma) in DMSO (0.5% f.c.) was added. Receptor surface levels at 37°C were monitored over time by FACS analysis after labeling with anti-CXCR4 (12G5) and anti-CXCR7 (9C4) and Alexa Fluor 488-F(ab′)_2_ goat anti-mouse IgG. In control experiments cells were incubated at 0°C with or without CXCL12 for 1 hour and washed with PBS or subjected to an acid wash. After acidic wash chemokines were fully removed and did not affect antibody binding.

### Chemokine Uptake and Degradation

MDCK cells expressing human CXCR7, CXCR7-DRYLAIV, CXCR4, mock-transfected MDCK, or primary HUVECs were grown on Transwell® insets (Costar, pore size 8 µm). When the cells reached confluence the culture medium was changed to Hank's balanced salt solution (HBSS) containing 10 mM HEPES, and 1% BSA. Radio-labeled [^125^I] CXCL12 (GE Healthcare, 0.25 nM) was added to the apical side of the monolayer for 3 hours at 37°C. To assess uptake and transport of ligand across the monolayer, both top and bottom contents of the Transwell as well as cell lysates were collected. Trichloracetic acid (TCA, 10% f.c.) precipitation was used to distinguish intact (precipitated) from degraded (TCA soluble) chemokine. The radioactivity of the samples was quantified using a gamma counter (Packard Instruments).

### Confocal Microscopy of MDCK-CXCR7 Monolayers

MDCK cells expressing human CXCR7 or CXCR4 were grown on glass bottom dishes. Confluent cells were incubated for 15 min with anti-CXCR7 (11G8 R&D) or anti CXCR4 (MAB173 R&D) in growth medium containing 0.1% saponin followed by incubation with Alexa Fluor 488-F(ab′)_2_ goat anti-mouse IgG in the same medium. After washing the secondary antibodies dishes were immediately analyzed with a Leica TCS SP5 confocal microscope.

To monitor ligand-independent antibody uptake, MDCK cells co-expressing membrane-anchored mCherry fluorescent protein and human CXCR7 or CXCR4 were grown on glass cover slips. When confluent, cells were incubated with anti-human CXCR7 (9C4) or anti-human CXCR4 (12G5) for 60 min at 37°C. Location of the primary antibody was revealed with Alexa Fluor 488-F(ab′)_2_ goat anti-mouse IgG after fixing for 5 min in 4% paraformaldehyde and permeabilizing the cells with 0.02% Tween20. Slides were washed with PBS-Tween20 (0.02%) and incubated for 30 min with Alexa Fluor 488-F(ab′)_2_ goat anti-mouse IgG, Alexa Fluor 594 phalloidin (both Invitrogen) and DAPI. Mounted slides were viewed with a Leica TCS SP5 confocal microscope.

### Receptor Re-Expression at the Cell Surface

MDCK cell and Daudi surface molecules were cleaved by proteinase K (0.1 mg/ml in PBS) for 3 hours on ice before transfer to PBS, 2% FBS, 2 mM EDTA, 50 µM cycloheximide. Receptor surface re-expression at 37°C was monitored over time by FACS analysis using antibodies recognizing the extracellular C-terminus of the transferrin receptor (OKT9), and the extracellular N-termini of CXCR4 (6H8) [24] and CXCR7 (9C4) [5] and secondary Alexa Fluor 488-F(ab′)_2_ goat anti-mouse IgG.

### mRNA Expression Constructs for Zebrafish

The construct used for global expression of CXCR7-EGFP was generated by cloning the *cxcr7-egfp* ORF into the pSP64TS vector that contains 5′ and 3′ UTRs of the *Xenopus globin* gene. Capped sense mRNA was synthesized using the mMessageMachine kit (Ambion) according to the manufacturer's instructions.

### Microinjections for Global Expression and Knockdown in Zebrafish Embryos

RNA and morpholino antisense oligonucleotides were microinjected into the yolk of one-cell stage embryos. For global expression of CXCR7 225 pg of *cxcr7-egfp-globin* mRNA were injected. Knockdown of both zebrafish CXCL12 versions was achieved using 0.4 pmol of oligonucleotides against *cxcl12a* (5′–TTGAGATCCATGTTTGCAGTGTGAA-3′), and *cxcl12b* (5′-TTGCTATCCATGCCAAGAGCGAGTG-3′)

### Spinning Disk Confocal Microscopy of Zebrafish Embryos

Confocal fluorescence images were obtained using a Zeiss AxioImager.M1 microscope fitted with a CascadeII camera (Photometrics) and VS-Laser Control, all controlled by VisiView software (Visitron Systems).

### Confocal Microscopy of Chemokine Uptake in Human Umbilical Cords and Mouse Hearts

Sections of 3–5 mm thickness of fresh human umbilical cords were placed in DMEM containing 1% human albumin, 200 nM fluorescent chemokine or PBS. When indicated, 10 µM AMD3100 (Sigma) or 1.5 µM CXCL11 were added to the medium. After 30 min incubation the sections were washed in DMEM and mounted in O.C.T. compound (VWR). Cryosections of 20 µm were fixed for 10 min in 4% paraformaldehyde, washed in PBST, and incubated for 1 h with anti-human CD31 (BD Biosciences) in PBST, 1% goat serum (Dako). Slides were washed with PBST and incubated for 1 h with Alexa Fluor 594-F(ab′)_2_ goat anti-mouse IgG (Invitrogen) and DAPI. Recombinant fluorescent chemokines were produced in the laboratory by expressing CXCL12 tagged at the C-terminus with fluorescent proteins in insect cells (E.C. and M.T., unpublished). Hearts of wild-type mice (BALB/c) were excised, and heart tips were removed to access the ventricles. After rinsing with DPBS, 2% FBS, Liquemin® (Roche) the specimens were incubated with fluorescent chemokine as described above. For confocal microscopy of the cryosections, nuclei of fixed and permeabilized cryosections were stained with DAPI.

### Statistical Analysis

All statistical analyses were performed using SigmaPlot software (Systat Software). Statistical comparisons between datasets were made with Student's t-test.

## Results

**Figure 1:**
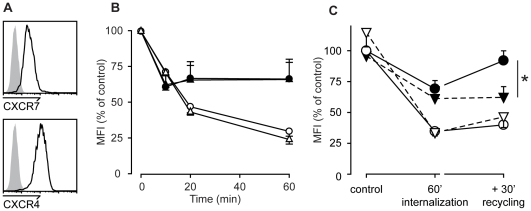
CXCL12 induced internalization of CXCR7 and CXCR4. (A) FACS analysis of resting Daudi B cells expressing endogenous CXCR4 and CXCR7. (B) Summary from three independent observations showing the time course of receptor expression of Daudi B cells expressing endogenous CXCR4 (open symbols) and CXCR7 (closed symbols) incubated with 100 nM (circles) or 1 µM (triangles) CXCL12. Extracellular bound chemokine was removed by an acidic wash (see Methods) and receptor surface expression was measured at the indicated times by FACS analysis. P<0.5 for ligand-induced internalization of CXCR7 and CXCR4. (C) Daudi B cells were incubated for 1 h at 37°C with 250 nM CXCL12. Extracellular bound chemokine was removed by an acidic wash and samples were split. Surface expression of CXCR7 (closed symbols) and CXCR4 (open symbols) was measured by FACS analysis directly (60′ internalization), or following an additional incubation for 30 min at 37°C to allow receptor reexpression (+30′ recycling). DMSO vehicle (circles) and 2 µM Bafilomycin A1 (triangles) were present during the entire procedure and a 60 min pre-treatment of the cells. MFIs are shown as percent of DMSO control without chemokine. Data from three independent observations; * P<0.05.

We previously reported that CXCL12-induced internalization of CXCR7 is inconsistent between primary circulating human CD19^+^ B cells and mouse pre 300.19 B cells stably expressing the receptor [10]. The chemokine induces CXCR7 internalization in transfected cells, but has little effect on the level of surface receptor expression on primary B cells. Similar lack of CXCR7 down-regulation after CXCL12 treatment was observed in other cells which express endogenous CXCR7, such as monocytes, the monocytic cell line THP-1, B cell clones and tumor derived cells. We now reiterated this observation in more detail using Daudi (Figure 1A) and Raji cells which express endogenous CXCR4 and CXCR7 at the plasma membrane [10]. Receptor surface expression was determined with specific monoclonal antibodies by FACS analysis. Bound chemokine, which could prevent antibody binding, was efficiently removed by an acidic wash prior antibody application [25]. In agreement with previous reports stimulation of the cells with CXCL12 induces a concentration-dependent disappearance of CXCR4 from the cell surface (not shown) [25], [26]. Figure 1B shows the maximum internalization of CXCR4 and CXCR7 which was induced by CXCL12 treatment. Approx. 50% of CXCR4 became internalized after 20 min and down-regulation increased to about 70–80% (75%±10% SD) after 1 h (Figure 1B). By contrast, little overall internalization (10–25%) of CXCR7 was detected after 60 min. However, at early time points after CXCL12 addition a transient CXCR7 down-regulation was observed which showed a maximum (up to 40%) after 10 min and recovered thereafter. The time course with a minimum at 10 min was consistently observed in Daudi and Raji cells (Figure 1B). The finding suggests that CXCR7 expression at the cell surface is tightly regulated and the receptor is rapidly replenished from intracellular stores after an initial agonist-induced internalization. Indeed CXCR7 expression fully recovered after removal of the chemokine and incubation at 37°C for 30 min. By contrast CXCR4 expression remained down regulated under these conditions (Figure 1C). Bafilomycin A is a well established inhibitor of vATPases, which mediates the gradual acidification of organelles going from the cell surface to lysosomes [27]–[29]. Treatment of Daudi cells with bafilomycin A had no effect on ligand-induced CXCR4 down regulation and CXCL12 induced CXCR7 internalization (Figure 1C). However, recycling of CXCR7 was markedly attenuated, suggesting that lowering the pH in the lumen of the endosome is required for the dissociation of CXCL12 from CXCR7 and thereby promoting subsequent receptor recycling to the plasma membrane. Receptor *de novo* synthesis during the course of the experiments was excluded by the presence of the translation inhibitor cycloheximide.

**Figure 2:**
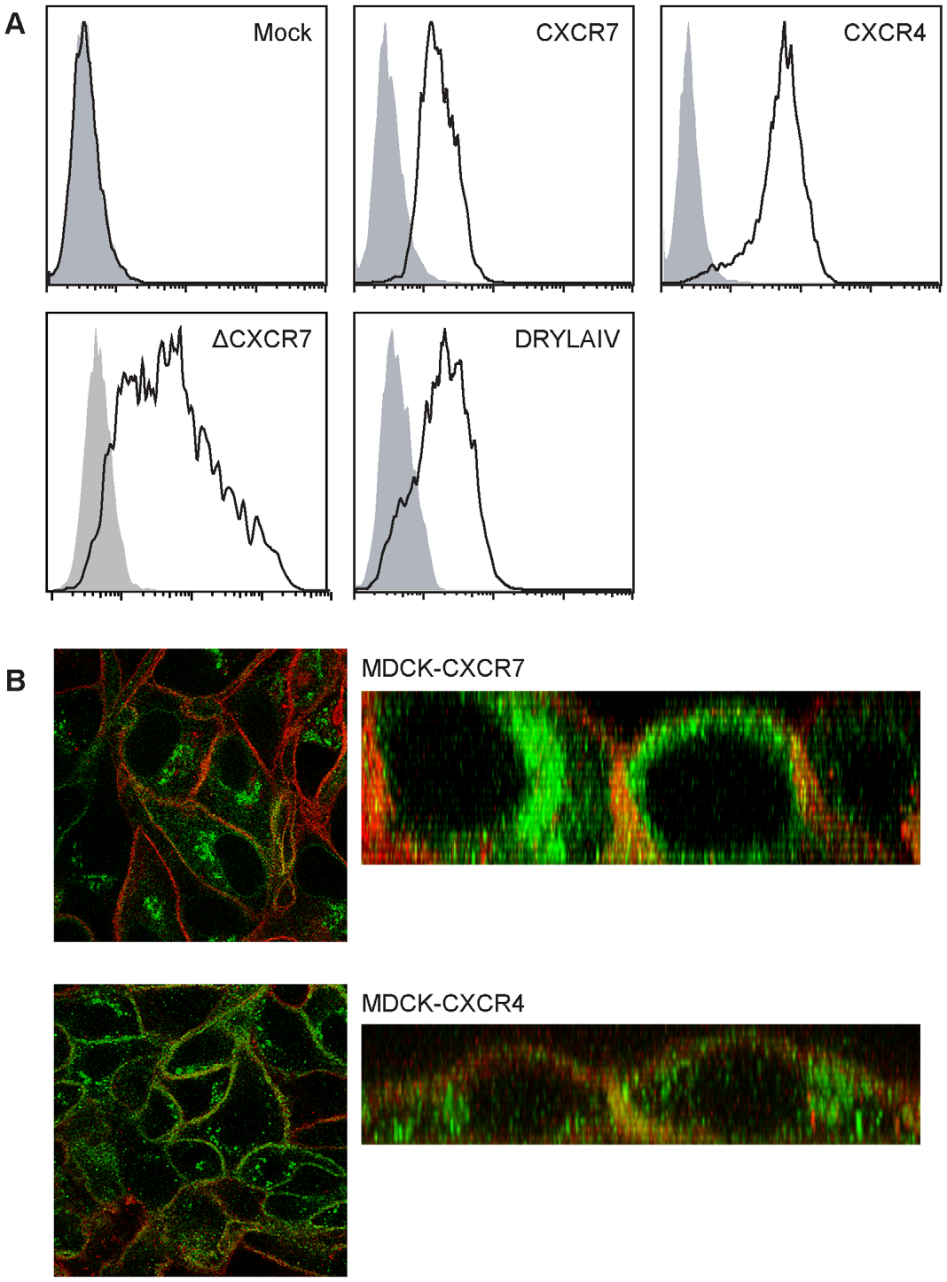
Ectopic expression of CXCR7 and CXCR4 on MDCK cells. (A) MDCK were stably transfected with empty vector (Mock), with CXCR7, CXCR4, a vector coding for a CXCR7 lacking the cytoplasmic C-terminus (ΔCXCR7), and a vector coding for a chimeric CXCR7 containing the DRYLAIV motive of CXCR4. Receptor expression was determined by FACS analysis using saturating antibody concentrations (see Methods). (B) Confocal immunofluorescence analysis of unfixed MDCK cells expressing CXCR7 (upper panels) or CXCR4 (lower panels). Cells also expressed N-ter-Lck mCherry as membrane marker (red fluorescence). Left panels: confocal images of planes cut through intracellular regions of MDCK monolayers. Right panels: x-z planes reconstructed from confocal x-y stacks. For receptor (green) detection anti-CXCR7 (11G8 R&D) or anti CXCR4 (MAB173 R&D) were used. Receptor-bound primary antibodies were revealed with goat anti mouse IgG conjugated with Alexa488 (green fluorescence).

We therefore hypothesized that CXCR7 acts as a scavenger for CXCL12. To this end Madin-Darby canine kidney (MDCK) epithelial cells were stably transfected with CXCR7, CXCR4, or CXCR7 lacking the cytoplasmic C terminus (ΔCXCR7). Because CXCR7 does not trigger pertussis toxin sensitive responses [8], we created also a chimeric receptor where we replaced the DRYLSIT-sequence in the second intracellular loop of CXCR7 with the corresponding DRYLAIV-sequence from CXCR4. This sequence is considered critical for functional coupling of GPCRs to G_i_-proteins [8], [30]. Restoration of the motif in the scavenger D6 confers weak ligand-induced signaling activity [31]. The expression of all receptors on the cell surface was confirmed by FACS analysis (Figure 2A). The chimeric CXCR7 was expressed to a similar level as its wild type counterpart (Figure 2A). Nevertheless, introduction of the canonical DRYLAIV motif into CXCR7 instead of DRYLSIT did not result in CXCL12-inducible chemotaxis, calcium mobilization, or ERK activation when expressed in either MDCK cells or 300.19 pre-B cells (not shown). Confocal microscopy of permeabilized MDCK cells revealed a marked punctuated staining arising from below the cell surface, suggesting that a large fraction of both receptors resides on intracellular compartments (Figure 2B).

**Figure 3:**
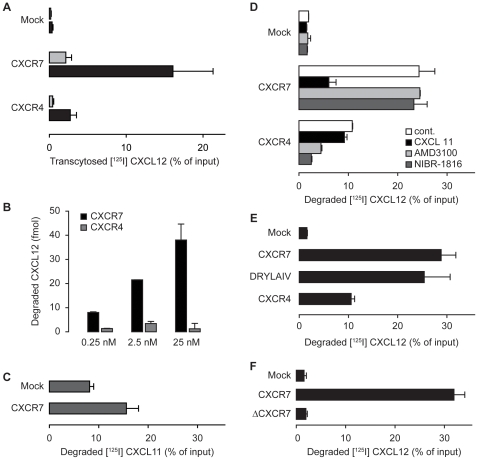
Degradation of CXCL12 mediated by CXCR7 and CXCR4. (A) MDCK cells stably transfected with empty vector (Mock), with CXCR7, and CXCR4 were incubated with 0.25 nM [^125^I]-CXCL12 for 3 h at 37°C added to the apical side. Black bars, TCA resistant radioactivity recovered from the lower part of the transwell; grey bars, radioactivity which was precipitated with TCA. Data from 5 experiments performed in triplicates. (B) Degradation of CXCL12 mediated by CXCR7 is not saturated by increasing concentrations of ligand. MDCK cells stably expressing CXCR7 or CXCR4 were incubated with increasing concentrations of [^125^I]-CXCL12 as in (A). Total TCA resistant counts were used to calculate the amount of degraded chemokine. Representative data shown from one experiment performed in triplicates. (C) MDCK stably transfected with empty vector (Mock) or CXCR7 were exposed to 0.25 nM [^125^I]-CXCL11 as in B. In three independent experiments more pronounced spontaneous degradation of CXCL11 in Mock-transfected cells compared to CXCL12 degradation was observed. (D) MDCK stably transfected with empty vector (Mock), with CXCR7, and CXCR4 were exposed to 0.25 nM [^125^I]-CXCL12 as in A in the presence of medium (cont.), 1 µM CXCL11 or the CXCR4 inhibitors AMD3100 (10 µM) and NIBR-1816 (10 µM). (E) The DYLAIV motif is not critical for CXCR7 activity. MDCK stably transfected with empty vector (Mock), with CXCR7, CXCR4 and a vector coding for a chimeric CXCR7 containing the DRYLAIV motive of CXCR4 were incubated as in B. Data from experiments that were performed in triplicates (n = 5, except DRYLAIV n = 2). (F) MDCK stably transfected with empty vector (Mock), with CXCR7, or a vector coding for a CXCR7 lacking the cytoplasmic C terminus (ΔCXCR7) were exposed to [^125^I]-CXCL12 as in B.

The scavenging activity of CXCR7 was measured using transfected MDCK cells seeded on Transwell® insets and grown to confluence. Radiolabeled [^125^I] CXCL12 was then added to the upper compartment and the cells incubated for 3 h at 37°C. To distinguish between degraded CXCL12 and intact or only partially proteolysed chemokine trichloroacetic acid (TCA) was added to the medium collected from the upper and lower compartment as well as the cell lysate to precipitate proteins. Radioactivity that is insensitive to TCA precipitation reflects chemokine that was cleaved by the cells during the incubation time. We compared the efficiency of MDCK cells expressing similar levels of CXCR7 and CXCR4 (Figure 2A) to degrade CXCL12. Cells expressing CXCR7 secreted significantly more CXCL12-derived radioactivity into the lower compartment than their CXCR4^+^ counterparts (Figure 3A). With either receptor the majority of the radioactivity was insensitive to TCA treatment, indicating that CXCL12 becomes largely degraded. Similar amounts of cleaved CXCL12 were also detected in the upper compartment of the transwell. We therefore summed all TCA-sensitive radioactivity recovered from the bottom of the well, in the cell lysate and from the upper well to calculate the total degradation activity (Figures 3B–F). We could not find evidence that the small fraction of TCA-sensitive radioactivity, which was transcytosed from the apical to the basal side by CXCR7, contained biological activity.

Over the concentration range tested (0.25 nM–25 nM CXCL12) CXCR7 mediated pronounced degradation of the chemokine as opposed to CXCR4. The difference of the two receptors in promoting the proteolysis was most evident at 25 nM CXCL12. Noteworthy, the reported binding affinity of CXCL12 for CXCR7 (Kd ∼ 0.2–0.4 nM) is considerably higher than for CXCR4 (Kd ∼ 2–4 nM) [5], [7], [32]. However, when the CXCL12 concentration in the assay was raised up to 100 times the Kd the capacity of CXCR7 to mediate degradation of the chemokine appeared not to be saturated, but rather increased linearly. By contrast, the effectiveness of CXCR4 to mediate proteolysis of CXCL12 showed a maximum at 2.5 nM and did not further augment at 25 nM (Figure 3B). The more prominent CXCR4 internalization shown in figure 1, contrasts the more efficient processing of CXCL12 by CXCR7. The apparent discrepancy is best explained with a scavenger function of CXCR7. In this model the receptor continuously cycles between the plasma membrane and an intracellular compartment, where the chemokine is delivered for degradation and CXCR7 returns to the membrane at the cell surface. In this way the receptor can efficiently clear CXCL12 from the external medium. Contrarily, CXCR4 upon ligand binding is internalized and becomes to a large extent degraded [25], [26], [33].

The second reported ligand for CXCR7 is CXCL11. Indeed, MDCK cells expressing CXCR7 degraded [^125^I] CXCL11 (Figure 3C) although with lower efficiency. Nevertheless, CXCL11 readily competed proteolysis of CXCL12 by CXCR7 (Figure 3D) and, as predicted from the known receptor specificity, CXCL11 had no effect on the moderate CXCR4 mediated CXCL12 degradation. Conversely, addition of a 100 fold excess of unlabelled CXCL12 expectedly attenuated the uptake and degradation of radiolabeled ligand of both receptors (not shown). The results underline specificity of CXCR7 to scavenge CXCL12. Two small molecule inhibitors of CXCR4, AMD3100 (10 µM) and NIBR-1816 (10 µM) [34], [35] markedly reduced the degradation by CXCR4, but had no effect on CXCR7 mediated scavenging of CXCL12. The data indicate further that binding and internalization of cognate chemokine by CXCR7 and CXCR4 are independent of each other.

**Figure 4:**
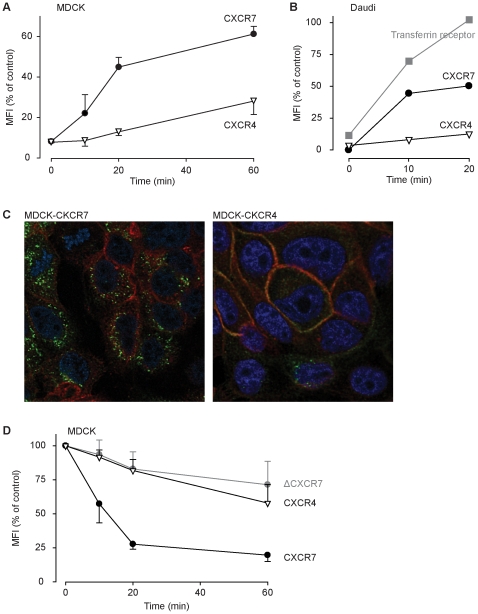
Ligand-independent receptor cycling. (A) MDCK cells expressing CXCR7 or CXCR4 were treated on ice with proteinase K to eliminate receptor epitopes from the surface. Cells were washed and incubated at 37°C in medium. At the indicated times aliquots were removed from the incubation and receptor surface expression measured by FACS analysis. (B) Daudi B cells expressing endogenous CXCR7, CXCR4, and transferrin receptor were treated with proteinase K as in (A), and subjected to FACS analysis. (C) MDCK cells expressing membrane-anchored mCherry (red color) and CXCR7 or CXCR4 were incubated with receptor specific monoclonal antibodies at 37°C for 30 min. Cells were fixed, permeabilized and stained with goat anti-mouse F(ab)_2_ Alexa 488. Red: Actin stained with Alexa Fluor 594 phalloidin; blue Nuclei stained with DAPI. Ligand-independent receptor internalization was visualized by confocal microscopy. Representative data shown from one experiment out of 5. (D) MDCK cells expression CXCR7, CXCR4, or a vector coding for a CXCR7 lacking the cytoplasmic C terminus (ΔCXCR7) were incubated on ice with receptor-specific antibodies. After removal of unbound antibodies cells were shifted to 37°C. At the indicated times aliquots were withdrawn and incubated with secondary goat anti-mouse F(ab)_2_ Alexa 488 at 0°C. Receptor surface expression was measured by FACS analysis.

Figure 3E shows that MDCK cells expressing CXCR7 when exposed to 0.25 nM [^125^I]-CXCL12 degraded approx. 30% of the added chemokine. By contrast, in cells transfected with an empty plasmid (mock) only marginal background proteolysis was observed. Cells carrying CXCR4 displayed a markedly reduced capacity to degrade CXCL12 compared with CXCR7. Exchanging the DRY-motif with the sequence of CXCR4 had no effect on CXCR7-mediated degradation, suggesting that this motif is not critical for the activity of the receptor. By contrast, deletion of the intracellular C-terminus (ΔCXCR7) completely abolished CXCL12 degradation mediated by CXCR7 (Figure 3F), suggesting that the domain is required for trafficking of the receptor. From the above experiments it is not clear if cycling of CXCR7 from the plasma membrane to endosomes is ligand induced or may also occur constitutively. To address this question we measured the dynamics of CXCR4 and CXCR7 expression at the plasma membrane in the absence of chemokines. Daudi cells or MDCK cells stably transfected with CXCR7 or CXCR4 were treated on ice with proteinase K. The protease is active at the low temperature, although with delayed kinetics, but membrane trafficking is completely abrogated [36]. Proteinase K cleaves the N-termini which contain the epitopes of CXCR7 for the mAb 9C4 [5] and of CXCR4 for the mAb 6H8 [24]. Following removal of the protease the cells were shifted to 37°C in the presence of cycloheximide. Re-emerging of the respective receptor epitopes was measured by FACS analysis. Figure 4A shows that in MDCK cells CXCR7 rapidly reappeared after shifting to 37°C reaching an expression level of 60% after one hour compared to that measured on untreated control cells. By contrast, after one hour of incubation at 37°C the immunoreactive CXCR4 was about 20% of control. In Daudi cells the reappearance after proteinase K treatment of endogenous CXCR4 and CXCR7 was compared with a typical ligand-independent cycling receptor, the transferrin receptor. After 20 min, the surface levels of the transferrin receptor were fully restored and expression of CXCR7 recovered by 50%. Conversely, during the same time CXCR4 levels reached only 20%. Together the findings suggest an intermediate cycling activity for CXCR7 which is less rapid compared with the transferrin receptor, but markedly faster than CXCR4, consistent with the more pronounced degradation of CXCL12 mediated by CXCR7.

Ligand independent cycling of CXCR7 was further investigated with antibody uptake experiments. MDCK cells expressing CXCR7 or CXCR4 were incubated for 1 h at 37°C in the absence of chemokines with specific monoclonal antibodies known to block receptor function (9C4 and 12G5, respectively) [5], [37]. Following removal of extracellular antibodies by stringent washing, cells were fixed and immunecomplexes detected with F(ab′)_2_ goat anti mouse IgG Alexa488 by confocal microscopy. In CXCR7 expressing MDCK cells a pronounced intracellular staining was observed which is associated with endosomal compartments. By contrast, CXCR4^+^ cells contained much less fluorescent endosomes (Figure 4C). The marked antibody uptake in CXCR7^+^ cells in the absence of stimulation suggest spontaneous receptor cycling between the plasma membrane and endosomal compartments. To further verify ligand-independent receptor cycling MDCK cells expressing CXCR4,CXCR7 or ΔCXCR7 were incubated with the respective antibodies at 0°C. Excess of antibody was removed and the cells shifted to 37°C in the presence of cycloheximide. Detection of the primary antibodies bound to surface-expressed receptors by FACS analysis revealed a rapid time dependent decrease of CXCR7 (Figure 4D). The loss of primary antibody from the cell surface is in agreement with ligand-independent internalization of CXCR7. Cells expressing ΔCXCR7 showed a marginal loss of primary antibody from the cells surface over time, in accordance with the notion that ΔCXCR7 does not internalize upon ligand exposure [5] and the failure of mediating CXCL12 degradation (Figure 3D). As expected primary antibody bound to CXCR4 expressed on the plasma membrane decreased only marginally consistent with the negligible spontaneous internalization. As both, ligand independent cycling and CXCL12-induced receptor internalization (Figure 1) can be observed for CXCR7 it is conceivable that the two mechanism work synergistically or at least act in parallel. As a whole our findings are in full agreement with the conclusion that CXCR7 expressed on MDCK cells acts as specific decoy for CXCL12 and CXCL11.

**Figure 5:**
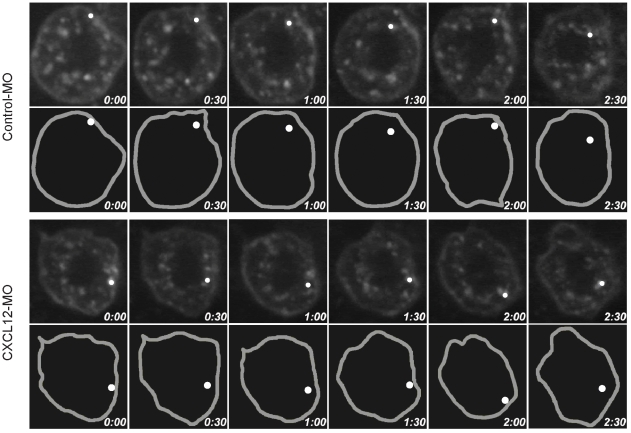
Ligand-independent cycling of CXCR7 in zebrafish embryos. In embryos expressing CXCR7-EGFP the receptor localizes at the plasma membrane (grey mark in lower panels) and intracellular vesicles (white dot). Time laps (time stamps) images shows that the vesicles are in contact with the membrane internalize and recycle back to the membrane. Internalization and cycling of CXCR7-EGF is similar in the presence (control–MO) or absence of CXCL12 (CXCL12–MO), underling the ligand independent mechanism. For the complete time laps see supplementary movies S1 cont and S2 CXCL12.

In zebrafish contradicting opinions on the role of CXCR7 have been reported, suggesting a signaling [17] versus a scavenger function [19]. To investigate this controversy, we followed the subcellular localization of the receptor in zebrafish cells. CXCR7-EGFP (CXCR7 tagged at the C-terminus with EGFP) was expressed in embryos alone or in combination with a morpholino oligonucleotide suppressing CXCL12 expression [19], [21]. Figure 5 shows frames from time-lapse movies (Supplementary Information, movies S1 (control - MO) and S2 (CXCL12-MO)) which document the spontaneous pinching off from the plasma membrane of CXCR7 containing endosomes and their re-association with the membrane. The movement of CXCR7 appears independent of the presence of CXCL12 indicating that CXCR7 can, in agreement with its proposed scavenger function, spontaneously cycle from and back to the plasma membrane in zebrafish embryonic cells. Boldajipour et al. [19] proposed that by removing CXCL12 at the posterior side CXCR7 sharpens the chemotactic gradient promoting CXCR4-dependent migration of primordial germ cells. The present observation provides an explanation for the efficiency of CXCR7 in supporting CXCR4-mediated migration of primordial germ cells and is consistent with the view that CXCR7 that is expressed by somatic cells functions as a scavenger.

**Figure 6:**
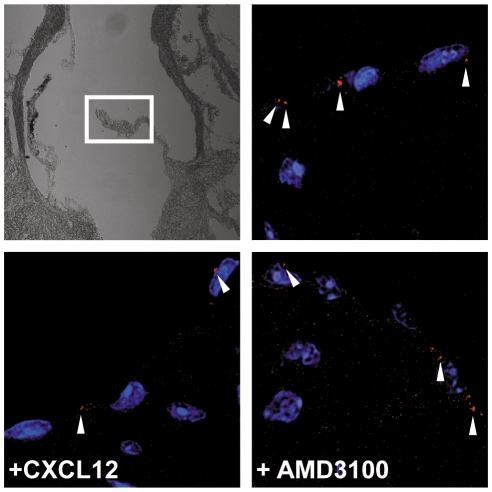
Uptake of CXCL12 by mouse heart valve. The aortic heart valves of wild type mice internalize CXCL12 tagged with a fluorescent protein (CXCL12-mCherry, upper right, red dots marked with arrowheads). Uptake of CXCL12-mCherry is partially competed with unlabeled 1.5 µM CXCL12 (lower left), and is partially resistant to administration of 10 µM AMD3100 (lower right). Pictures were taken in the indicated area (white frame upper left).

Targeted deletion of *Cxcr7* leads to perinatal death with a marked cardiac phenotype that is associated with septum and heart valve malformation. The phenotype is also manifest upon conditional deletion of *Cxcr7* in endothelium [9]. A possible explanation for the phenotype could be that lack of CXCR7 gives rise to CXCL12-stimulated CXCR4-dependent hyperplasia. We therefore tested if mouse heart valves can scavenge CXCL12. Hearts were removed from wild type laboratory (BALB/c) mice and cut transversally. The opened hearts which included all valves were incubated with 150 nM CXCL12 tagged at the C-terminus with the fluorescent protein mCherry. Frozen sections of the hearts reveal endosomes in valve endothelial cells loaded with CXCL12-mCherry. Uptake was marginally impaired in the presence of 10 µM AMD3100, suggesting that the process is mediated by CXCR7 rather than by CXCR4. In line with this a recent report indicates that AMD3100 at 10 µM slightly affects CXCL12 binding to CXCR7 [38]. Addition of 1.5 µM untagged CXCL12 expectedly competed the uptake of CXCL12-mCherry (Figure 6). The results are consistent with a scavenger activity of CXCR7 on valve endothelium in adult mice.

**Figure 7:**
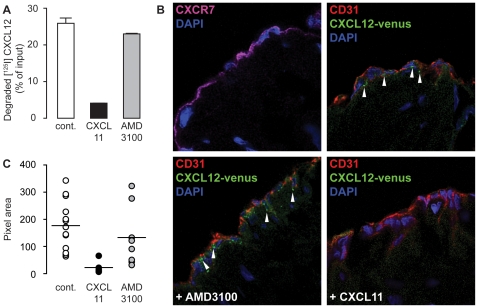
Scavenging activity of CXCR7 in primary human endothelium. (A) Human umbilical vein endothelial cells (HUVECs) were exposed for 3 h at 37°C to [^125^I]-CXCL12 in the presence of 1 µM CXCL11 or 10 µM of the CXCR4 inhibitor AMD3100. TCA resistant counts represent degraded chemokine and were plotted as percent of total added counts (cont). (B) Vein endothelial cells in fresh human umbilical cord slices express CXCR7 (purple upper left, blue nuclei DAPI), vein endothelial cells (CD31 marker red) internalize CXCL12 tagged with a fluorescent protein (CXCL12-venus, upper right, green dots marked with arrowheads), uptake of CXCL12-venus is not affected by 10 µM AMD3100 (lower left), but is competed with 1.5 µM CXCL11 (lower right). (C) Several images (512×512 pixels) from 3 independent experiments as shown in (B) were analyzed using Metamorph software. Total pixel areas showing fluorescence of the CXCL12-venus per image are shown. P<0.05 between cont. and CXCL11, not significant between cont. and AMD3100.

Human umbilical cord endothelial cells (HUVECs) express CXCR4 and CXCR7 [7], [39]. To test the scavenging activity of CXCR7 expressed on these cells, early passage HUVECs were incubated with [^125^I]-CXCL12 for 3 h at 37°C. Figure 7A shows that HUVECs proteolyzed nearly 30% of the added chemokine. Degradation was marginally inhibited by AMD3100 (10 µM), but efficiently competed by 1 µM CXCL11, suggesting proteolysis is largely attributed to CXCR7-dependent scavenging. CXCR7 is expressed on human vascular endothelium and is further upregulated by inflammatory cytokines such as TNFα or IL-1β [7], [40]. To reveal a potential scavenging activity of CXCR7 on primary human tissue we incubated fresh human umbilical cord slices with CXCL12 tagged at the C-terminus with fluorescent protein Venus. Figure 7B (upper left) shows the expression of CXCR7 at the lining endothelium of the umbilical veins. Incubation of the slices with 200 nM CXCL12-Venus causes the appearance of fluorescent endosomes, suggesting the uptake of the chemokines by surface receptors (upper right). Pretreatment of the slices with 10 µM AMD3100 for 10 min had no effect on the uptake. By contrast, addition of 1.5 µM CXCL11 fully competed the uptake of CXCL12. The data are in accordance with the observations made with HUVECs and substantiate the conclusion that CXCR7 acts as scavenger on human endothelial cells. Figure 7C summarizes data obtained with umbilical cords from different donors. Although a notable donor to donor variation is visible, the statistical analysis reveals CXCL11-sentive uptake of CXCL12, which is marginally affected by AMD3100, supporting the view that CXR7 acts as a scavenger in endothelial cells.

## Discussion

The tight regulation of the surface expression of CXCR7 in the presence of CXCL12 suggests that the receptor functions a scavenger. Biochemical analysis in MDCK cells and HUVECs reveal an effective capacity of CXCR7 to eliminate CXCL12 from its surrounding environment. The efficiency of scavenger receptors depends on their capability to mediate the elimination of a stoichiometric multiplicity of ligands from the surrounding environment. In figure 3B we demonstrate that degradation of CXCL12 by CXCR7 is not saturated even at elevated chemokine concentrations.

Heptahelical receptors have been described which bind chemokines with high affinity, but fail to couple to heterotrimetric G-proteins and to induce typical responses. In mammalians three ‘atypical’ chemokine receptors were characterized namely the Duffy antigen receptor for chemokines (DARC), D6, and CCX-CKR (CCR11). DARC has recently been shown to transcytose inflammatory chemokines from the basal to the luminal site of the venular endothelium. Since chemokines remain bound to DARC expressed in the lumen of venules the receptor supports chemokine migratory activity and leukocyte extravasation [41]. On erythrocytes DARC is assumed to function as sink or reservoir for many inflammatory chemokines [42]. The primary function of D6 and CCX-CKR is to act as scavenger for chemokines. D6 contributes to the resolution of inflammation by internalizing and destroying inflammatory CC chemokines [43], [44]. CCX-CKR effectively internalizes the homeostatic CC chemokines CCL19 and CCL21 targeting them for degradation [45]. However, CXCL12 does not bind to any of the ‘atypical’ receptors.

Like the other decoy receptors for chemokines CXCR7 does not activate heterotrimeric G-proteins, a compulsory step for typical signal transduction of heptahelical receptors [8], [46]. Nevertheless, internalization of this class of receptors does not require coupling to G-proteins. Recently it was reported that CXCL12 induces arrestin binding to CXCR7, indicating that the agonist can promote internalization through clathrin coated pits [38]. In line with this observation we found that deletion of the C-terminus of CXCR7 markedly reduces scavenger activity (Figure 3D and 4D).

Exceptionally among chemokine receptors CXCR4 can induce the sustained activation of the PI 3-kinase/PKB and MAPK cascade [46]. This responsiveness may contribute to the proliferative and pro-survival characteristics of the CXCL12/CXCR4 axis [47], [48]. Depending on the context, however, the properties can be detrimental for the host organism und must therefore be under tight control. The prolonged signaling by CXCR4 demands the continuous presence of CXCL12 at the cell surface and is abrogated when the chemokines becomes degraded, e.g. by CD26/dipeptidyl peptidase IV [26], [49], [50]. Blocking of the CXCL12/CXCR4 signaling axis leads to the proliferation of primitive hematopoietic stem cells [51] while excessive signaling by CXCR4 accounts for the molecular pathoetiology of the WHIM syndrome [52] and is associated with tumor growth and metastasis formation [53]. These observations indicate that the level and availability of CXCL12 for CXCR4 is tightly regulated under normal conditions and the present data indicate that CXCR7, which is widely expressed, may critically contribute to this regulation.

An alternative regulation of CXCR4 activity by CXCR7 could be potential heterodimerization of the receptors. Co-transfection of CXCR7 and CXCR4 in HEK cells indicates that the receptors can functionally heterodimerize [9], [15]. However, in primary human CD19^+^ B cells, which express both receptors in circulation, co-internalization, a hall mark of heptahelical receptor dimers, was not observed, suggesting that heterodimerization in primary cells is of minor relevance [1], [10].

The perinatal lethality of mice with a deletion of *Cxcr7* [9], [54] could be explained by the lack of CXCL12 scavenging in these animals. Platelets which carry large amounts of CXCL12 may deploy the chemokine when undergoing mechanical stress in the heart circulation. The excessive presence of the chemokine may subsequently stimulate CXCR4 which is highly expressed on the heart endothelium (MT, unpublished), leading to aberrant proliferation [55]. Such view is also in line with the expression of CXCR7 on the heart valves of adult mice. The evolutionary conserved mechanism of CXCL12 scavenging by CXCR7 further supports the importance of this activity [19]. In a previous study, when CXCR7 as receptor for CXCL12 was not yet disclosed, scavenging of CXCL12 by endothelial CXCR4 was proposed, which was partially attenuated in the presence of CXCR4 inhibitors. The authors further suggested that CXCR4 translocates CXCL12 to the bone marrow [56]. Whether CXCR7 can transcytose CXCL12, in analogy to the activity of DARC [41], remains to be established. The present findings, however, fully support the conclusion that the principal function of CXCR7 is to act as scavenger for CXCL12.

## Supporting Information

Movie S1 Cont Ligand-independent cycling of CXCR7 in zebrafish embryos. In embryos expressing CXCR7-EGFP the receptor localizes at the plasma membrane (grey) and intracellular vesicles (blue dot). Time laps shows that the vesicles are in contact with the membrane internalize and recycle back to the membrane. Internalization and cycling of CXCR7-EGF in the presence (control - MO) of CXCL12.
(4.61 MB MOV)

Movie S2 CXCL12 Ligand-independent cycling of CXCR7 in zebrafish embryos. In embryos expressing CXCR7-EGFP the receptor localizes at the plasma membrane (grey) and intracellular vesicles (blue dot). Time laps shows that the vesicles are in contact with the membrane internalize and recycle back to the membrane. Internalization and cycling of CXCR7-EGF in the absence of CXCL12 (CXCL12 - MO).
(3.27 MB MOV)
